# ArchSpiral: An iPad-Based Digital Archimedean Spiral Assessment for Objective Motor Evaluation in Rare Diseases

**DOI:** 10.64898/2026.07.27.26358872

**Published:** 2026-07-30

**Authors:** Daniel R. Schecter, Rory J. Tinker, Matteo Danieletto, Georgia MacDonald, Tamas Kozicz, Eva Morava, Benjamin S Glicksberg

## Abstract

**Objectives:**

We developed and evaluated ArchSpiral, an iPad application that digitizes the International Cooperative Ataxia Rating Scale (ICARS) spiral tracing task to address the need for portable rare disease assessment while preserving clinical scoring and generating quantitative digital biomarkers.

**Materials and Methods:**

ArchSpiral (Swift/SwiftUI/PencilKit) captures Apple Pencil or finger tracings and computes tracing accuracy, root mean squared error, duration, average speed, pen lifts, pauses, steadiness, and an automated ICARS-compatible morphology score. Twelve participants with congenital disorders of glycosylation completed paired paper-digital assessments.

**Results:**

Digital ICARS scores spanned the scoring range (1–4). Paper and digital scores agreed exactly in 11 of 12 assessments (linear weighted κ = 0.91). Steadiness showed the strongest association with paper-based ICARS scores (ρ = −0.82, FDR-adjusted *P* = 0.008).

**Discussion:**

ArchSpiral enables standardized digital spiral assessment, while preserving compatibility with conventional ICARS scoring and adding objective quantitative measures for longitudinal rare disease research and clinical care.

## Full Text Availability

The license terms selected by the author(s) for this preprint version do not permit archiving in PMC. The full text is available from the preprint server.

